# Clinical Factors Associated with Inappropriate Antibiotic Use in Children with Acute Bronchiolitis

**DOI:** 10.3390/children12101303

**Published:** 2025-09-26

**Authors:** Jung-Woo Rhim, Jin Lee, Minsung Kim, Seung Beom Han, Hwan Soo Kim, Soo Young Lee

**Affiliations:** Department of Pediatrics, College of Medicine, The Catholic University of Korea, Seoul 06591, Republic of Korea; jwrhim@catholic.ac.kr (J.-W.R.); pedleejin@catholic.ac.kr (J.L.); msturtle73@catholic.ac.kr (M.K.); pedhskim@catholic.ac.kr (H.S.K.); sylee@catholic.ac.kr (S.Y.L.)

**Keywords:** acute bronchiolitis, antibiotics, fever, C-reactive protein

## Abstract

**Highlights:**

**What are the main findings?**

- Most children with acute bronchiolitis received antibiotic therapy despite the very low prevalence of concurrent serious bacterial infections.
- Fever and elevated C-reactive protein levels were significantly associated with antibiotic prescription.

**What is the implication of the main finding?**

- Clinician education is needed to address the lack of rationale supporting antibiotic use in children with acute bronchiolitis.

**Abstract:**

Background/Objectives: Although serious bacterial infections are rare in children with acute bronchiolitis, which is predominantly caused by respiratory viruses, antibiotics are often prescribed. This study aimed to identify clinical factors associated with inappropriate antibiotic use in children with acute bronchiolitis. Methods: We retrospectively reviewed the medical records of 612 children aged 3 to 23 months who were hospitalized with their first episode of acute bronchiolitis. Based on antibiotic prescription at admission, children were categorized into two groups: the antibiotic group and the non-antibiotic group. Clinical variables were compared between groups to identify factors associated with inappropriate antibiotic use. Results: Of the 612 children included, 514 (84.0%) received antibiotic therapy on admission. Bacteremia was diagnosed in 0.5% of the cohort. In multivariate analysis, fever (*p* = 0.002) and C-reactive protein ≥0.50 mg/dL (*p* < 0.001) were independently associated with inappropriate antibiotic use. The duration of fever after admission was significantly longer in the antibiotic group than in the non-antibiotic group (*p* = 0.005). Conclusions: Despite the very low prevalence of serious bacterial infections and lack of clinical benefit, most children hospitalized with acute bronchiolitis received antibiotic therapy. Inappropriate antibiotic use was primarily driven by fever and elevated C-reactive protein levels, which lacked sufficient diagnostic justification.

## 1. Introduction

Acute bronchiolitis is the most common lower respiratory tract infection resulting in hospitalization among infants [1]. As acute bronchiolitis is primarily viral in origin, most typically due to respiratory syncytial virus (RSV), international clinical guidelines discourage routine antibiotic use in affected children [1]. However, inappropriate antibiotic use remains common. Globally, approximately 25% of children with acute bronchiolitis receive antibiotics [1], and in Korea, more than half of infants receive antibiotic therapy [2]. Inappropriate antibiotic use is a major cause of increasing antibiotic resistance, resulting in increased morbidity and mortality at the individual level, as well as broader disease transmission and rising healthcare and economic burdens globally [3]. Therefore, optimizing antibiotic use is a global public health priority.

A recent nationwide study using the National Health Insurance claims data in Korea identified several factors associated with inappropriate antibiotic use in children with acute bronchiolitis [2]. Antibiotics were prescribed more frequently in children aged 12–23 months than in infants aged <12 months, and in those who were hospitalized, living in non-capital regions, treated in primary or secondary hospitals, or managed by non-pediatricians compared with their respective counterparts [2]. However, clinical symptoms and signs were not analyzed in that study [2], and only a few studies have investigated clinical predictors for inappropriate antibiotic use at the individual patient level [4,5]. Identifying modifiable clinical factors driving inappropriate antibiotic use is essential for developing targeted antimicrobial stewardship programs.

This study aimed to assess the clinical impact of antibiotic therapy and to identify clinical factors associated with inappropriate antibiotic use in children hospitalized with acute bronchiolitis. The findings may guide interventional strategies to improve antibiotic stewardship for pediatric respiratory viral infections.

## 2. Materials and Methods

### 2.1. Subjects and Study Design

We retrospectively reviewed the medical records of children aged between 3 and 23 months who were admitted to Bucheon St. Mary’s Hospital (Gyeonggi-do, Republic of Korea) between September 2015 and August 2024 with a primary diagnosis of acute bronchiolitis. Among them, children who experienced their first episode of acute bronchiolitis were included in this study. We excluded those who developed respiratory symptoms ≥2 days after admission (suggesting hospital-acquired infection), those who began antibiotic therapy ≥2 days after admission, and those for whom initial antibiotic therapy was clinically appropriate. Demographic, clinical, and laboratory data were collected retrospectively. Clinical factors included history of preterm birth; underlying diseases; family history of allergic diseases; vital signs, presenting symptoms, chest examination findings, and antibiotic use on admission; fever duration; and clinical severity including oxygen supplementation, mechanical ventilator care, intensive care unit (ICU) admission, and in-hospital death. Laboratory factors included testing results obtained on admission such as complete blood counts and serum levels of C-reactive protein (CRP), blood urea nitrogen, creatinine, sodium, potassium, chloride, aspartate transaminase, alanine transaminase, blood and urine cultures, and urinalysis. When available, culture results from other normally sterile body fluids were investigated. Culture studies were performed at the discretion of the attending physicians, and specimens for cultures were obtained on the admission date, prior to initiation of antibiotic therapy when antibiotics were administered. Chest X-ray findings were assessed based on radiologist interpretations.

The included children were divided into two groups based on antibiotic therapy at admission: the antibiotic group and non-antibiotic group. We compared the investigated factors between the two groups to identify clinical factors associated with antibiotic use. The occurrence of serious bacterial infections (SBIs) was also assessed. In children who presented with fever on admission, the duration of fever after admission was compared between the two groups to determine the clinical impact of antibiotic use. In those who were afebrile on admission, the incidence of new-onset fever during hospitalization was analyzed. As a subgroup analysis, the study period was divided into the pre-COVID-19 period (September 2015 to August 2020) and the post-COVID-19 period (September 2020 to August 2024). For each period, the same comparative analyses between the antibiotic and non-antibiotic groups were performed. This study was approved by the Institutional Review Board of Bucheon St. Mary’s Hospital, and the requirement for informed consent was waived due to the retrospective design (approval number: HC25RISI0001, approval date: 7 January 2025).

### 2.2. Definitions

Acute bronchiolitis was clinically diagnosed in children younger than 24 months who presented with respiratory symptoms (e.g., cough, rhinorrhea, tachypnea, or dyspnea) together with abnormal auscultation findings (e.g., wheezing, rales, rhonchi, or decreased breath sounds). Antibiotic use on admission was considered appropriate when it was prescribed for concurrent diagnoses likely to represent bacterial infections, such as acute otitis media (AOM), cervical lymphadenitis, or skin and soft tissue infection. Empirical antibiotic therapy for children with pyuria was also deemed appropriate regardless of the final urine culture results. Pyuria was defined as ≥10 white blood cells per high-power field on flow cytometry of unspun urine samples. SBIs included bacteremia and growth of pathogenic bacteria from cultures of normally sterile body fluids. Bacteremia was diagnosed when pathogens were isolated from blood cultures. The following organisms were considered skin contaminants and not classified as bacteremia: coagulase-negative staphylococci, *Bacillus* spp. other than *Bacillus anthracis*, *Corynebacterium* spp., *Cutibacterium acnes*, *Lactobacillus* spp., *Micrococcus* spp., and viridans streptococci [6]. Since children with pyuria were excluded from this study, urinary tract infection (UTI) was not considered for SBIs.

### 2.3. Statistical Analysis

Categorical factors were compared between the antibiotic and non-antibiotic groups using a chi-square test, while continuous factors were compared using a Mann–Whitney U test due to non-normal distributions across all continuous factors. Factors showing significant differences in the univariate analysis were included in the multivariate analysis using a binary logistic regression test to identify independent factors associated with antibiotic use. For multivariate analysis, continuous factors were dichotomized using optimal cut-off values for predicting antibiotic use, which were determined by receiver operating characteristic (ROC) curve analysis. Annual antibiotic prescription rates were compared using the linear-by-linear association test. All statistical analyses were performed using the R Statistical Software (v4.3.3, R Core Team 2024, R Foundation for Statistical Computing, Vienna, Austria). Cases with incomplete records for relevant analyses were excluded. Statistical significance was set at *p*-value < 0.05.

## 3. Results

**Figure 1:**
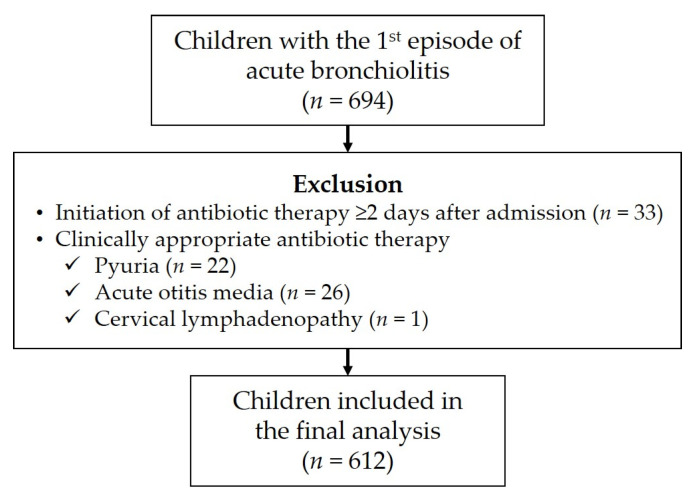
Flowchart of patient selection and exclusion.

A total of 694 children were hospitalized with their first episode of acute bronchiolitis during the study period. Among them, 33 (4.8%) children who began antibiotic therapy ≥2 days after admission were excluded. In addition, 26 (3.7%) children with a concurrent AOM, 22 (3.2%) with pyuria, and one (0.1%) with cervical lymphadenitis on admission were excluded. The remaining 612 children were included in the study analysis (Figure 1).

### 3.1. Clinical Factors Associated with Antibiotic Therapy

**Figure 2:**
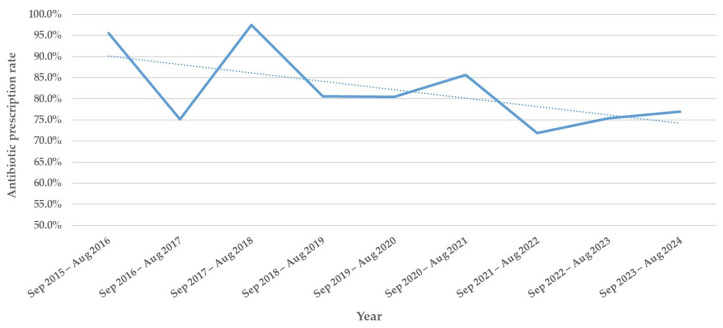
Annual antibiotic prescription rates during the study period.

Of the 612 children, 514 (84.0%) started antibiotic therapy on admission (median duration: 4 days, range: 1–26). The antibiotic prescription rate was significantly lower in the post-COVID-19 period than in the pre-COVID-19 period (75.2% vs. 85.9%, *p* = 0.006). Annual prescription rates had already begun to decline significantly before the COVID-19 pandemic (*p* = 0.003, Figure 2). Children in the antibiotic group were significantly older (*p* = 0.030) and more frequently presented with fever (*p* < 0.001) and decreased breath sounds (*p* = 0.037) than those in the non-antibiotic group (Table 1).

Laboratory findings revealed that the antibiotic group had significantly higher neutrophil counts (*p* < 0.001) and CRP level (*p* < 0.001) but lower lymphocyte (*p* = 0.002) and eosinophil (*p* = 0.026) counts compared with the non-antibiotic group (Table 2). Abnormal chest X-ray findings were also more frequent in the antibiotic group (*p* = 0.043, Table 2). Although hemoglobin levels; platelet counts; and blood urea nitrogen, serum creatinine, aspartate transaminase, alanine transaminase, and electrolyte levels differed statistically between the groups (Table 2), these values largely remained within normal ranges and were not considered clinically significant.

Accordingly, multivariate analysis included age, fever on admission, decreased breath sounds, neutrophil count, CRP level, and abnormal chest X-ray findings. ROC curve analysis identified the following optimal cut-off values for continuous factors predicting antibiotic use: age ≥8 months, neutrophil count ≥ 3000/mm^3^, and CRP ≥ 0.50 mg/dL. In the multivariate analysis, fever on admission (*p* = 0.002) and CRP ≥ 0.50 mg/dL (*p* < 0.001) were identified as independent predictors of antibiotic use in children with acute bronchiolitis (Table 3).

In the pre-COVID-19 period (*n* = 503), children in the antibiotic group were significantly older (*p* < 0.001) and more frequently presented with fever (*p* < 0.001) and diarrhea (*p* = 0.037) as well as higher neutrophil counts (*p* < 0.001) and CRP levels (*p* < 0.001), compared with the non-antibiotic group (Table S1). In the multivariate analysis, fever on admission (*p* < 0.001) and CRP ≥ 0.50 mg/dL (*p* < 0.001) remained independent predictors of antibiotic use, consistent with the findings in the overall study population (Table S2). In the post-COVID-19 period (*n* = 109), children in the antibiotic group were significantly older (*p* < 0.001) and had higher neutrophil counts (*p* < 0.001) compared with the non-antibiotic group, whereas the frequencies of fever and CRP levels were comparable between the two groups (Table S3). In the multivariate analysis, a higher neutrophil count was an independent predictor of antibiotic use (*p* = 0.002, Table S4).

### 3.2. Clinical Impact of Antibiotic Therapy

Blood cultures were obtained in 600 (98.0%) of the 612 children, and bacterial growth was observed in 28 (4.7%). Among the 600 children who underwent blood cultures, 95 (15.8%) did not receive antibiotic therapy, and five (17.9%) of the 28 children with positive blood cultures also did not receive antibiotic therapy. Bacteremia was diagnosed in only three (0.5%) children: two with *Moraxella* spp. and one with *Pseudomonas aeruginosa*. All three received antibiotic therapy, and all cases occurred during the pre-COVID-19 period. The remaining 25 (4.2%) isolates were skin contaminants. Hospitalization duration was significantly longer in the antibiotic group than in the non-antibiotic group (*p* = 0.003, Table 1). Among 402 children with fever on admission, the duration of fever after admission was longer in the antibiotic group (*p* = 0.005, Table 1). Among the 210 children who were afebrile on admission, new-onset fever during hospitalization occurred more frequently in the antibiotic group than in the non-antibiotic group, although the difference was not significant (Table 1). Of the 552 children who did not require oxygen therapy on admission, the proportion who subsequently required oxygen therapy during hospitalization was similar between the two groups: 14 of 462 (3.0%) in the antibiotic group versus 3 of 90 (3.3%) in the non-antibiotic group.

## 4. Discussion

In this study, we investigated clinical factors associated with antibiotic use in children hospitalized with acute bronchiolitis. Although antibiotics are not recommended for treatment of acute bronchiolitis, 84.0% of our cohort received such therapy. Fever and elevated CRP levels on admission were independently associated with inappropriate antibiotic use.

Previous studies have similarly found that fever, elevated CRP, older age, and increased clinical severity are associated with antibiotic use in children with acute bronchiolitis [4,5,7]. Clinicians in our study may interpret fever and elevated inflammatory markers as potential indicators of bacterial infection, leading to empirical antibiotic therapy. However, both fever and CRP have limited reliability in distinguishing bacterial from viral infection. Fever occurred in up to 90% of hospitalized children with acute bronchiolitis in whom viral pathogens were identified [8], and was present in 65.7% of our cohort on admission. Despite this, 84.5% of the included children received antibiotics, suggesting that elevated CRP levels may have been an additional driver of antibiotic use. Although some studies have reported associations between elevated CRP levels and bacterial pneumonia, the proposed thresholds were ≥4 or ≥5 mg/dL [9,10]. In our cohort, only 2.8% and 5.1% of children had CRP levels ≥5 and ≥4 mg/dL, respectively. Therefore, the lower cut-off of ≥0.5 mg/dL identified in our study is unlikely to be a reliable parameter for guiding antibiotic use. Inflammatory markers other than CRP, such as procalcitonin, may be useful for predicting bacterial infections and guiding appropriate antibiotic use. However, research on procalcitonin has primarily focused on specific populations, such as children admitted to the ICU, and its clinical utility in acute bronchiolitis remains unclear [11,12]. Encouragingly, during the post-COVID-19 period, the overall antibiotic prescription rate decreased significantly compared with the pre-COVID-19 period, and fever and elevated CRP levels were no longer independent predictors for antibiotic use. However, given that this subgroup represented only 17.8% of the study population, these findings should be interpreted with caution and confirmed by further studies.

Consequently, in current clinical practice, antibiotic therapy in children with acute bronchiolitis largely depends on identification of a causative pathogen. Although respiratory multiplex polymerase chain reaction (PCR) testing is now widely used, its impact on reducing antibiotic use is limited, as shown by a recent meta-analysis [13]. This finding was reaffirmed in a recent randomized clinical trial assessing the use of multiplex PCR as a point-of-care test in children presenting with fever and/or respiratory symptoms [14]. Similarly, multiplex PCR testing in hospitalized children with acute bronchiolitis did not effectively reduce inappropriate antibiotic use [4]. Therefore, we should make efforts to limit unnecessary antibiotic therapy based on the clinical context, particularly the rare incidence of concurrent SBIs in children with acute bronchiolitis, rather than based on laboratory test results. In our cohort, bacteremia was identified in only 0.5% of children, and no other types of SBI were concurrently diagnosed although the prevalence of UTI was not assessed. Previous studies reported the prevalence of UTI and bacteremia in children with acute bronchiolitis to be 0.2–0.8% and 0.1–0.4%, respectively [4,15,16,17]. These findings underscore that only a small proportion of children diagnosed with acute bronchiolitis are likely to benefit from empirical antibiotic therapy.

Inappropriate antibiotic use not only increases antibiotic resistance and healthcare costs but may also have long-term health consequences, such as allergic diseases, obesity, and juvenile idiopathic arthritis, particularly in children exposed to antibiotics prior to two years of age [18]. This emphasizes the need for greater caution when prescribing antibiotics for acute bronchiolitis, which primarily affects infants. Notably, children who received antibiotics in our study had longer durations of hospitalization and fever after admission compared with those who did not. Similar associations have also been reported in previous studies [4,7]. Although factors such as the infecting virus or variations in host immune responses might have influenced fever and hospitalization durations, our findings indicated that antibiotic use provided no clinical benefit in children with viral infections.

This study has several limitations. First, it was conducted in a single secondary referral hospital and included only hospitalized children, limiting generalizability. However, many children with acute bronchiolitis are managed in outpatient or primary care settings with milder disease, likely resulting in even lower rates of SBIs and need for antibiotics. Second, as a retrospective study, important clinical factors may have been missed, and unmeasured influences such as physician judgment or parental expectations for antibiotics could not be assessed. Third, although concurrent bacterial pneumonia has been reported in some studies of acute bronchiolitis [19,20], we did not consider this diagnosis in our analysis. Those studies primarily focused on ICU patients with a greater proportion of them showed consolidations on chest X-ray suggestive of bacterial pneumonia [19,20]. In contrast, a recent Korean study reported significantly lower bacterial identification rates in children diagnosed with bronchiolitis/bronchitis than in those with pneumonia [21]. In that study, only *Mycoplasma pneumoniae* was identified in 3.5% of children with bronchiolitis/bronchitis, of whom 8.4% had consolidations on chest X-ray [21]. In our cohort, only 0.8% of children exhibited consolidation, suggesting that bacterial lower respiratory infections were rare.

## 5. Conclusions

In conclusion, our findings revealed a clear gap between current clinical practice and evidence-based guidelines, as reflected by the high rate of antibiotic use in children with acute bronchiolitis. Inappropriate antibiotic therapy provided no clinical benefit in these patients. Inappropriate antibiotic therapy was primarily driven by fever and elevated CRP levels, which were not sufficient for clinical justification. These results highlighted the need for improved clinician education as part of antimicrobial stewardship programs. Future prospective multicenter studies are required to validate these findings and ultimately improve appropriate antibiotic use for childhood viral infections.

## Figures and Tables

**Table 1:** Comparison of clinical factors between the antibiotic and non-antibiotic groups.

Factor	Total (*n* = 612)	Antibiotic Group (*n* = 514)	Non-Antibiotic Group (*n* = 98)	*p*-Value
Age, months, median (range)	8 (3–23)	8 (3–23)	7 (3–23)	<0.001
Sex				0.331
Male	392 (64.1)	325 (63.2)	67 (68.4)	
Female	220 (35.9)	189 (36.8)	31 (31.6)	
Hospital days, median (range)	5 (2–12)	5 (2–12)	4 (2–11)	0.003
Preterm birth ^1^	84 (17.3)	70 (17.2)	14 (17.7)	0.911
Underlying disease	57 (9.3)	51 (9.9)	6 (6.1)	0.236
Congenital heart disease	15 (2.5)	14 (2.7)	1 (1.0)	0.486
Allergic disease	12 (2.0)	10 (1.9)	2 (2.0)	1.000
Neurodevelopmental disease	12 (2.0)	9 (1.8)	3 (3.1)	0.419
Urogenital disease	6 (1.0)	6 (1.2)	0 (0.0)	0.597
Respiratory disease	13 (2.1)	13 (2.5)	0 (0.0)	0.241
Family history of allergy	49 (8.0)	38 (73.9)	11 (11.2)	0.200
Symptoms on admission				
Fever	402 (65.7)	364 (70.8)	38 (38.8)	<0.001
Cough	608 (99.3)	510 (99.2)	98 (100.0)	1.000
Sputum	548 (89.5)	457 (88.9)	91 (92.9)	0.242
Rhinorrhea	525 (85.8)	436 (84.8)	89 (90.8)	0.120
Dyspnea	136 (22.2)	115 (22.4)	21 (21.4)	0.837
Vomiting	121 (19.8)	103 (20.0)	18 (18.4)	0.703
Diarrhea	49 (8.0)	38 (7.4)	11 (11.2)	0.200
Seizures	4 (0.7)	3 (0.6)	1 (1.0)	0.503
Skin rash	3 (0.5)	2 (0.4)	1 (1.0)	0.408
Fever onset after admission ^2^	34 (16.2)	28 (18.7)	6 (10.0)	0.124
Vital signs on admission				
Heart rate > 150/minute	58 (9.5)	49 (9.5)	9 (9.2)	0.914
Respiratory rate > 60/minute	3 (0.5)	3 (0.6)	0 (0.0)	1.000
SpO_2_ < 90% ^3^	6 (1.1)	6 (1.4)	0 (0.0)	0.596
Chest examination findings				
Wheezing	535 (87.4)	449 (87.4)	86 (87.8)	0.913
Rales	327 (53.4)	276 (53.7)	51 (52.0)	0.763
Rhonchi	34 (5.6)	30 (5.8)	4 (4.1)	0.487
Decreased breathing sounds	23 (3.8)	23 (4.5)	0 (0.0)	0.037
Chest wall retractions	156 (25.5)	127 (24.7)	29 (29.6)	0.309
Fever days after admission, median (range) ^4^	1 (0–7)	1 (0–7)	0 (0–2)	0.005
Clinical severity				
Oxygen therapy	77 (12.6)	66 (12.8)	11 (11.2)	0.658
Mechanical ventilation	1 (0.2)	1 (0.2)	0 (0.0)	1.000
Receiving intensive care	2 (0.3)	2 (0.4)	0 (0.0)	1.000

SpO_2_: oxygen saturation measured by pulse oximeter. ^1^ Birth history was recorded for 486 children (407 in the antibiotic group and 79 in the non-antibiotic group). ^2^ This was evaluated in 210 children (150 in the antibiotic group and 60 in the non-antibiotic group) who did not present with fever on admission. ^3^ SpO_2_ was checked in 532 children (443 in the antibiotic group and 89 in the non-antibiotic group). ^4^ This was evaluated in 402 children (364 in the antibiotic group and 38 in the non-antibiotic group) who presented with fever on admission.

**Table 2:** Comparison of laboratory and radiological factors between the antibiotic and non-antibiotic groups.

Factor	Total (*n* = 612)	Antibiotic Group (*n* = 514)	Non-Antibiotic Group (*n* = 98)	*p*-Value
WBC count,/mm^3^, median (range)^1^	10,840 (3060–32,580)	10,860 (3060–32,580)	10,060 (4740–20,400)	<0.001
neutrophils	3506 (73–27,367)	3748 (73–27,367)	2276 (489–11,606)	<0.001
lymphocytes	5457 (888–15,088)	5353 (1229–14,059)	6195 (888–15,088)	<0.001
eosinophils	106 (0–2669)	97 (0–2669)	137 (0–1264)	<0.001
Hb, g/dL, median (range) ^1^	11.8 (7.2–15.1)	11.8 (7.2–15.1)	12.0 (8.3–14.2)	<0.001
PLT count,/mm^3^, median (range) ^1^	361,000	360,000	368,000	<0.001
	(107,000–1,076,000)	(107,000–1,076,000)	(183,000–583,000)	
CRP, mg/dL, median (range) ^2^	0.45 (0.01–19.60)	0.59 (0.01–19.60)	0.11 (0.01–1.84)	<0.001
BUN, mg/dL, median (range) ^2^	8.7 (1.6–21.2)	8.9 (2.5–21.2)	7.8 (1.6–17.8)	<0.001
Cr, mg/dL, median (range) ^2^	0.26 (0.10–0.46)	0.26 (0.10–0.46)	0.24 (0.17–0.43)	<0.001
AST, U/L, median (range) ^2^	37 (15–269)	37 (15–269)	39 (23–134)	<0.001
ALT, U/L, median (range) ^2^	21 (2–303)	21 (2–303)	24 (12–111)	<0.001
Na, mEq/L, median (range) ^2^	139 (133–144)	139 (133–144)	139 (134–144)	<0.001
K, mEq/L, median (range) ^2^	4.8 (3.3–6.2)	4.8 (3.3–6.2)	4.8 (3.3–6.0)	<0.001
Cl, mEq/L, median (range) ^2^	103 (93–110)	103 (93–110)	103 (99–109)	<0.001
Bacteremia ^3^	3 (0.5)	3 (0.6)	0 (0.0)	1.000
Radiological findings				
Normal lung fields	477 (77.9)	393 (76.5)	84 (85.7)	0.043
Bronchial infiltrates	135 (22.1)	109 (21.2)	14 (14.3)	0.117
Hyperinflation	25 (4.1)	24 (4.7)	1 (1.0)	0.094
Segmental/lobar consolidation	5 (0.8)	5 (1.0)	0 (0.0)	1.000

WBC: white blood cell; Hb: hemoglobin; PLT: platelet; CRP: C-reactive protein; BUN: blood urea nitrogen; Cr: creatinine; AST: aspartate transaminase; ALT: alanine transaminase. ^1^ A complete blood count was performed in 511 children in the antibiotic group and 98 in the non-antibiotic group. ^2^ CRP and blood chemistry tests were conducted in 513 children in the antibiotic and 98 in the non-antibiotic group. ^3^ Blood cultures were performed in 505 children in the antibiotic group and 95 in the non-antibiotic group.

**Table 3:** Multivariate analysis for independent factors associated with inappropriate antibiotic therapy.

Factor	Odds Ratio	95% Confidence Interval	*p*-Value
Age ≥ 8 months	1.19	0.74–1.90	0.482
Fever on admission	2.19	1.34–3.59	0.002
Decreased breathing sounds ^1^	5,520,121.83	NC	0.984
Neutrophil count ≥ 3000/mm^3^	1.54	0.93–2.56	0.096
CRP ≥ 0.50 mg/dL	3.78	1.99–7.16	<0.001
Chest X-ray abnormality	1.23	0.65–2.36	0.526

NC: not calculable. ^1^ The confidence interval could not be calculated due to the small number of events.

## Data Availability

The raw data supporting the conclusions of this article will be made available by the authors on request.

## Supplementary Materials

The following supporting information can be downloaded at: https://www.mdpi.com/article/10.3390/children12101303/s1, Table S1: Comparison of clinical factors between the antibiotic and non-antibiotic groups during the pre-COVID-19 period, Table S2: Multivariate analysis for independent factors associated with inappropriate antibiotic therapy during the pre-COVID-19 period, Table S3: Comparison of clinical factors between the antibiotic and non-antibiotic groups during the post-COVID-19 period, Table S4: Multivariate analysis for independent factors associated with inappropriate antibiotic therapy during the post-COVID-19 period.

## Abbreviations

The following abbreviations are used in this manuscript:

RSV	Respiratory syncytial virus
ICU	Intensive care unit
CRP	C-reactive protein
SBI	Serious bacterial infection
AOM	Acute otitis media
UTI	Urinary tract infection
ROC	Receiver operating characteristic
PCR	Polymerase chain reaction
