# Supplementary material for: Clinical Factors Associated with Inappropriate Antibiotic Use in Children with Acute Bronchiolitis
Source: Children (Basel). 2025 Sep 26;12(10):1303. doi: 10.3390/children12101303 (PMC12564678; doi:10.3390/children12101303)
Supplement: Supplementary file 1 [file children-12-01303-s001.zip › children-3864390-supplementary.pdf]

Table S1. Comparison of clinical factors between the antibiotic and non-antibiotic groups during the pre-COVID-19 period

Factor	Antibiotic group (*n* = 432)	Non-antibiotic group (*n* = 71)	*p*-Value
Age, months, median (range)	8 (3-23)	6 (3-22)	<0.001
Sex			0.121
Male	269 (62.3)	51 (71.8)	
Female	163 (37.7)	20 (28.2)	
Hospital days, median (range)	5 (2-12)	4 (2-11)	<0.001
Preterm birth[1]	59 (16.9)	6 (10.7)	0.241
Underlying disease	43 (10.0)	5 (7.0)	0.439
Congenital heart disease	14 (3.2)	1 (1.4)	0.707
Allergic disease	9 (2.1)	2 (2.8)	0.659
Neurodevelopmental disease	7 (1.6)	2 (2.8)	0.370
Urogenital disease	5 (1.2)	0 (0.0)	1.000
Respiratory disease	10 (2.3)	0 (0.0)	0.371
Family history of allergy	29 (6.7)	8 (11.3)	0.173
Symptoms on admission			
Fever	311 (72.0)	24 (33.8)	<0.001
Cough	428 (99.1)	71 (100.0)	1.000
Sputum	387 (89.6)	66 (93.0)	0.379
Rhinorrhea	363 (84.0)	64 (90.1)	0.183
Dyspnea	88 (20.4)	13 (18.3)	0.688
Vomiting	92 (21.3)	14 (19.7)	0.763
Diarrhea	34 (7.9)	11 (15.5)	0.037
Seizures	2 (0.5)	1 (1.4)	0.367
Skin rash	2 (0.5)	1 (1.4)	0.367
Fever onset after admission[2]	26 (21.5)	6 (12.8)	0.196
Vital signs on admission			
Heart rate >150/minute	45 (10.4)	6 (8.5)	0.611
Respiratory rate >60/minute	1 (0.2)	0 (0.0)	1.000
SpO_2 <90%[3]	5 (1.4)	0 (0.0)	1.000
Chest examination findings			
Wheezing	378 (87.5)	65 (91.5)	0.329
Rales	240 (55.6)	41 (57.7)	0.730
Rhonchi	25 (5.8)	1 (1.4)	0.154
Decreased breathing sounds	20 (4.6)	0 (0.0)	0.094
Chest wall retractions	101 (23.4)	19 (26.8)	0.536
Fever days after admission, median (range)[4]	1 (0-7)	1 (0-2)	0.011
Clinical severity			
Oxygen therapy	44 (10.2)	7 (9.9)	0.933
Mechanical ventilation	1 (0.2)	0 (0.0)	1.000
Receiving intensive care	2 (0.5)	0 (0.0)	1.000
WBC count, /mm^3, median (range)[5]	10,840 (3,060-32,580)	10,590 (4,740-20,400)	<0.001
neutrophils	3,641 (73-27,367)	2,390 (489-11,606)	<0.001
lymphocytes	5,388 (1,295-14,059)	6,163 (888-15,088)	<0.001
eosinophils	98 (0-2,669)	162 (0-806)	<0.001
Hb, g/dL, median (range)[5]	11.8 (7.2-15.1)	12.0 (8.3-14.2)	<0.001
PLT count, /mm^3, median (range)[5]	353,000 (107,000-1,076,000)	375,000 (183,000-583,000)	<0.001
CRP, mg/dL, median (range)[6]	0.59 (0.01-17.80)	0.11 (0.01-1.53)	<0.001
BUN, mg/dL, median (range)[6]	8.6 (2.5-21.2)	7.5 (1.6-14.2)	<0.001
Cr, mg/dL, median (range)[6]	0.26 (0.10-0.46)	0.24 (0.17-0.43)	<0.001
AST, U/L, median (range)[6]	37 (17-269)	38 (23-134)	<0.001
ALT, U/L, median (range)[6]	21 (2-303)	24 (12-111)	<0.001
Na, mEq/L, median (range)[6]	139 (133-144)	139 (134-144)	<0.001

K, mEq/L, median (range)[6]	4.8 (3.3-6.2)	4.8 (3.3-6.0)	<0.001
Cl, mEq/L, median (range)[6]	103 (93-110)	103 (99-109)	<0.001
Bacteremia[7]	3 (0.7)	0 (0.0)	1.000
Radiological findings			
Normal lung fields	328 (75.9)	59 (83.1)	0.184
Bronchial infiltrates	97 (22.5)	12 (16.9)	0.293
Hyperinflation	18 (4.2)	1 (1.4)	0.497
Segmental/lobar consolidation	2 (0.5)	0 (0.0)	1.000

SpO_2: oxygen saturation measured by pulse oximeter; WBC: white blood cell; Hb: hemoglobin; PLT: platelet; CRP: C-reactive protein; BUN: blood urea nitrogen; Cr: creatinine; AST: aspartate transaminase; ALT: alanine transaminase.

[1] Birth history was recorded for 349 children in the antibiotic group and 56 in the non-antibiotic group.

[2] This was evaluated in 121 children in the antibiotic group and 47 in the non-antibiotic group, who did not present with fever on admission.

[3] SpO_2 was checked in 366 children in the antibiotic group and 65 in the non-antibiotic group.

[4] This was evaluated in 311 children in the antibiotic group and 24 in the non-antibiotic group, who presented with fever on admission.

[5] A complete blood count was performed in 429 children in the antibiotic group and 71 in the non-antibiotic group.

[6] CRP and blood chemistry tests were conducted in 431 children in the antibiotic and 71 in the non-antibiotic group.

[7] Blood cultures were performed in 424 children in the antibiotic group and 69 in the non-antibiotic group.

Table S2. Multivariate analysis for independent factors associated with inappropriate antibiotic therapy during the pre-COVID-19 period

**Factor**	**Odds ratio**	**95% confidence interval**	***p*-Value**
Age ≥8 months	1.37	0.78-2.42	0.273
Fever on admission	2.80	1.56-5.01	<0.001
Diarrhea	0.49	0.22-1.10	0.085
Neutrophil count ≥3,000/mm^3	1.31	0.73-2.34	0.367
CRP ≥0.50 mg/dL	5.13	2.25-11.72	<0.001

CRP: C-reactive protein.

Table S3. Comparison of clinical factors between the antibiotic and non-antibiotic groups during the post-COVID-19 period

**Factor**	**Antibiotic group (*n* = 82)**	**Non-antibiotic group (*n* = 27)**	***p*-Value**
Age, months, median (range)	10 (3-23)	10 (3-22)	<0.001
Sex			0.390
Male	56 (68.3)	16 (59.3)	
Female	26 (31.7)	11 (40.7)	
Hospital days, median (range)	4 (2-9)	4 (3-10)	<0.001
Preterm birth[1]	11 (19.0)	8 (34.7)	0.130
Underlying disease	8 (9.8)	1 (3.7)	0.447
Congenital heart disease	0 (0.0)	0 (0.0)	NA
Allergic disease	1 (1.2)	0 (0.0)	1.000
Neurodevelopmental disease	2 (2.4)	1 (3.7)	1.000
Urogenital disease	1 (1.2)	0 (0.0)	1.000
Respiratory disease	3 (3.7)	0 (0.0)	0.573
Family history of allergy	9 (11.0)	3 (11.1)	1.000
Symptoms on admission			
Fever	53 (64.6)	14 (51.9)	0.237
Cough	82 (100.0)	27 (100.0)	NA
Sputum	70 (85.4)	25 (92.6)	0.510
Rhinorrhea	73 (89.0)	25 (92.6)	0.728
Dyspnea	27 (32.9)	8 (29.6)	0.750
Vomiting	11 (13.4)	4 (14.8)	1.000
Diarrhea	4 (4.9)	0 (0.0)	0.570
Seizures	1 (1.2)	0 (0.0)	1.000
Skin rash	0 (0.0)	0 (0.0)	NA
Fever onset after admission[2]	2 (6.9)	0 (0.0)	1.000
Vital signs on admission			
Heart rate >150/minute	4 (4.9)	3 (11.1)	0.361
Respiratory rate >60/minute	2 (2.4)	0 (0.0)	1.000
SpO_2 <90%[3]	1 (1.3)	0 (0.0)	1.000
Chest examination findings			
Wheezing	71 (86.6)	21 (77.8)	0.358
Rales	36 (43.9)	10 (37.0)	0.531
Rhonchi	5 (6.1)	3 (11.1)	0.406
Decreased breathing sounds	3 (3.7)	0 (0.0)	0.573
Chest wall retractions	26 (31.7)	10 (37.0)	0.610
Fever days after admission, median (range)[4]	1 (0-4)	0 (0-1)	0.532
Clinical severity			
Oxygen therapy	22 (26.8)	4 (14.8)	0.204
Mechanical ventilation	0 (0.0)	0 (0.0)	NA
Receiving intensive care	0 (0.0)	0 (0.0)	NA
WBC count, /mm^3, median (range)	11,405 (4,530-16,960)	8,810 (5,460-16,960)	<0.001
neutrophils	4,337 (226-24,372)	1,525 (718-7,691)	<0.001
lymphocytes	4,935 (1,229-11,394)	6,248 (1,026-12,991)	<0.001
eosinophils	92 (0-1,107)	127 (9-1,264)	<0.001
Hb, g/dL, median (range)	11.9 (9.3-13.7)	12.2 (11.2-13.3)	<0.001
PLT count, /mm^3, median (range)	399,000 (169,000-874,000)	357,000 (191,000-571,000)	<0.001
CRP, mg/dL, median (range)	0.63 (0.03-19.60)	0.16 (0.03-1.84)	0.921
BUN, mg/dL, median (range)	9.9 (3.4-18.5)	8.9 (2.9-17.8)	<0.001
Cr, mg/dL, median (range)	0.27 (0.17-0.43)	0.24 (0.17-0.42)	<0.001
AST, U/L, median (range)	35 (15-90)	41 (25-96)	<0.001
ALT, U/L, median (range)	19 (6-129)	21 (15-71)	<0.001
Na, mEq/L, median (range)	139 (134-143)	139 (136-141)	<0.001

K, mEq/L, median (range)	4.8 (3.6-6.1)	4.8 (3.9-5.9)	<0.001
Cl, mEq/L, median (range)	103 (96-109)	105 (101-106)	<0.001
Bacteremia[5]	0 (0.0)	0 (0.0)	NA
Radiological findings			
Normal lung fields	65 (79.3)	25 (92.6)	0.149
Bronchial infiltrates	17 (20.7)	2 (7.4)	0.149
Hyperinflation	6 (7.3)	0 (0.0)	0.333
Segmental/lobar consolidation	3 (3.7)	0 (0.0)	0.573

NA: not available; SpO_2: oxygen saturation measured by pulse oximeter; WBC: white blood cell; Hb: hemoglobin; PLT: platelet; CRP: C-reactive protein; BUN: blood urea nitrogen; Cr: creatinine; AST: aspartate transaminase; ALT: alanine transaminase.

[1] Birth history was recorded for 58 children in the antibiotic group and 23 in the non-antibiotic group.

[2] This was evaluated in 29 children in the antibiotic group and 13 in the non-antibiotic group, who did not present with fever on admission.

[3] SpO_2 was checked in 77 children in the antibiotic group and 24 in the non-antibiotic group.

[4] This was evaluated in 53 children in the antibiotic group and 14 in the non-antibiotic group, who presented with fever on admission.

[5] Blood cultures were performed in 81 children in the antibiotic group and 26 in the non-antibiotic group.

Table S4. Multivariate analysis for independent factors associated with inappropriate antibiotic therapy during the post-COVID-19 period

Factor	Odds ratio	95% confidence interval	*p*-Value
Age	1.01	0.93-1.09	0.860
Neutrophil count	4.75	1.81-12.48	0.002
